# Distribution of Capsular Types of *Campylobacter jejuni* Isolates from Symptomatic and Asymptomatic Children in Peru

**DOI:** 10.4269/ajtmh.18-0994

**Published:** 2019-08-05

**Authors:** Jesús D. Rojas, Nathanael D. Reynolds, Brian L. Pike, Nereyda M. Espinoza, Janelle Kuroiwa, Vihasi Jani, Paul A. Ríos, Rosa G. Nunez, Pablo P. Yori, Manuela Bernal, Rina A. Meza, Margaret N. Kosek, Andrea J. McCoy, Mark P. Simons, Michael J. Gregory, Patricia Guerry, Frédéric M. Poly

**Affiliations:** 1 Bacteriology Department, Naval Medical Research Unit-6 (NAMRU-6), Lima, Peru;; 2 Facultad de Ciencias Biológicas, Universidad Nacional Mayor de San Marcos, Lima, Peru;; 3 U.S. National Medical Research Center, Singapore;; 4 Enteric Diseases Department, Infectious Diseases Directorate, Naval Medical Research Center, Silver Spring, Maryland;; 5 Division of Infectious Diseases and International Health, University of Virginia, Charlottesville, Virginia

## Abstract

*Campylobacter jejuni* is the leading bacterial cause of diarrhea worldwide. A capsular polysaccharide (CPS) conjugate vaccine is under development and requires determination of the valency. However, distribution of CPS types circulating globally is presently poorly described. We aimed to determine whether CPS type distribution in Peru differs from that in other endemic regions. We used a multiplex polymerase chain reaction (PCR) assay for the detection of CPS encoding genes capable of distinguishing all 35 CPS types on *Campylobacter* isolates in two prospective communities based studies conducted in cohorts of children less than 59 months of age in Peru. Results showed that CPS type HS4 complex was the most prevalent, followed by HS3 complex and HS15. Differences in CPS type for symptomatology were not statistically significant. Most subjects demonstrated repeated infections over time with different CPS types, suggesting that CPS types may confer of a level of homologous protective immunity. In this dataset, some differences in CPS type distribution were observed in comparison to other low-middle income countries. Further studies need to be conducted in endemic areas to increase our knowledge of CPS type distribution and guide vaccine development.

## INTRODUCTION

*Campylobacter jejuni* is the leading bacterial cause of human diarrheal disease in both industrialized settings and settings of extreme poverty.^1,2^ Furthermore, a recent report by the WHO states that *Campylobacter* spp. are responsible for more than 96 million illnesses worldwide, including Guillain–Barré syndrome (GBS), a flaccid paralysis sequelae attributed to *C. jejuni* infections.^1^ In addition, developing countries are particularly afflicted by *C. jejuni* infections, with the pediatric population being most susceptible to this pathogen.^3^ Strikingly, despite its importance in global health, relatively little is known about virulence factors of this enteric pathogen.^4,5^ In vitro, CadF, FlpA, and JlpA have been identified as proteins that play a critical role in adherence of *Campylobacter* to HEp2 human epithelial cells.^6^ In addition, lipooligosaccharides, capsule, and flagella are recognized as playing a key role in the infectious process in INT407 and Caco-2 human epithelial cells.^7–10^ In the last decade, capsular polysaccharide (CPS) has been shown to be a determinant for serum resistance, invasion, adherence, and colonization of the human cell lines Caco-2 and INT-407^7^ and for modulation of host immune response.^11^

Both the Global Enterics Multi-Center Study^12^ and the Etiology, Risk Factors, and Interactions of Enteric Infections and Malnutrition and the Consequences for Child Health and Development Project (MAL-ED) study,^13^ two large multisite studies designed to determine the prevalence and impact of enteric infections in the developing world, have demonstrated the importance of *Campylobacter* as a cause of diarrhea in infants and young children. The MAL-ED study and a prior study have also demonstrated that *Campylobacter* infection is associated with linear growth deficits, even in the absence of GBS. Therefore, infection may have durable consequences on the well-being of children^14,15^ in addition to being a considerable risk factor for diarrhea in the early years of life.^16^

In recent years, a capsule-based vaccine has been developed and tested in a nonhuman primate model, the owl monkey, *Aotus nancymaae*,^17–19^ and is presently under phase I testing.^19^ To be feasible, given an apparent lack of heterologous protection, a CPS-based vaccine approach would need to target the most prevalent and pathogenic CPS types among *C*. *jejuni* isolates causing diarrhea. Nonetheless, the required valency for such vaccine is yet to be determined because of the lack of data from endemic regions.^20^ Thus, surveillance studies focused on key antigenic determinants are needed to understand the requirements and plausibility of vaccine development.^4,19,20^

Capsular polysaccharide typing of *C. jejuni* by means of Pennerʼs scheme has become one of the most widely accepted assays for determining serotypes of *C*. *jejuni*. Studies on the distribution of serotypes have been conducted since the development of the assay, but most of them were performed in developed countries.^20^ However, few studies describing CPS type distribution have been conducted in developing countries, where this microorganism is hyperendemic.^4,20^ The complexity of the Penner assay and the need for specific serological reagents limit the utility of this assay and may partially account for the lack of serotype data from low-middle income countries.^20^ More recently, a multiplex PCR has been described for the determination of CPS type by molecular means.^21,22^ Importantly, this assay is not adversely affected by phase variation of the CPS, which is problematic for traditional serotyping assays.^7^

**Figure 1.**
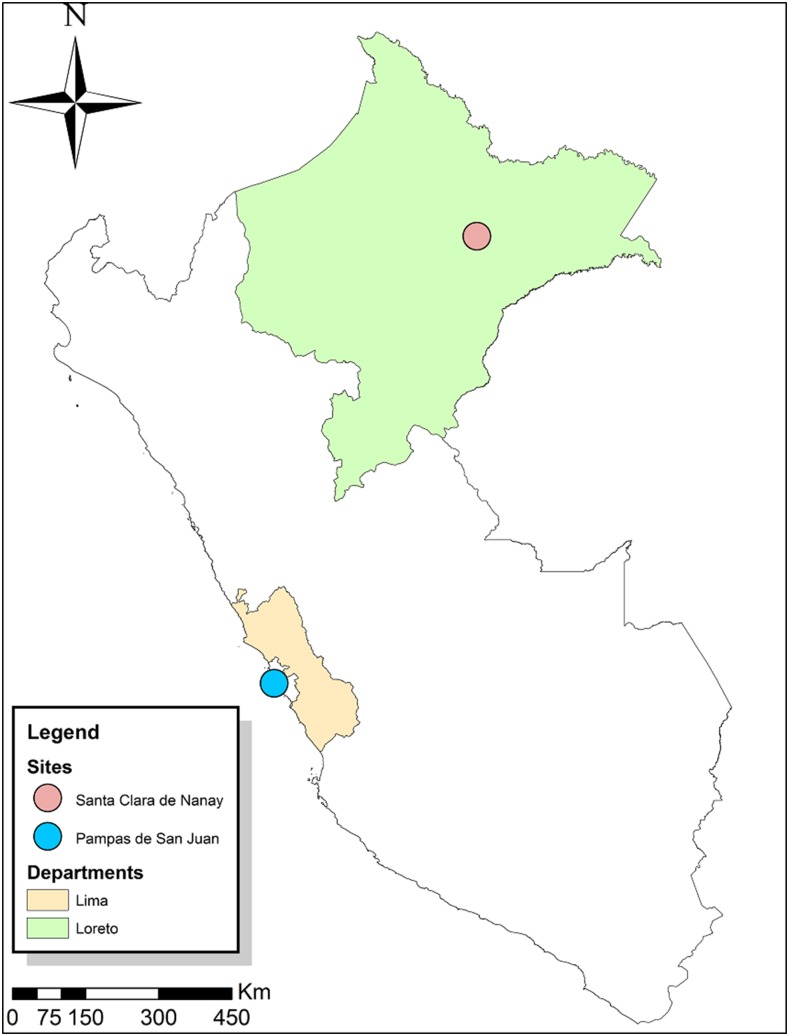
Location of study sites in Peru (ArcGIS Desktop: Release 10.0).

We sought to evaluate the relative distribution of CPS types and the potential association of specific CPS types with clinical disease in *C*. *jejuni* isolates from fecal samples obtained from children younger than 5 years in two different geographical areas in Peru: Santa Clara de Nanay, a rural community located in the northern Peruvian Amazon, 15 km southeast from the city of Iquitos,^15,23,24^ and Pampas de San Juan, a peri-urban shantytown on the Peruvian west coast near Lima (Figure 1).^25,26^

## METHODS

### Population.

*Campylobacter jejuni* isolates were obtained from two independent cohort studies for diarrhea in children younger than 72 months. Both sites similarly had limited access to basic sanitation conditions, and the potential for fecal contamination from human and animal sources was relatively high.^25,27^

The first study was conducted for 18 months in Pampas de San Juan, a peri-urban shantytown in Lima, located in the coastal region of Peru.^25,26^ Stool samples were collected monthly from children younger than 5 years by trained personnel to determine bacterial carriage state and additional cultures were obtained when liquid stools were observed. Carriage state is defined as an asymptomatic patient who is positive for *C. jejuni* isolation from a stool culture. A total of 378 *Campylobacter* isolates obtained from stool samples of 99 subjects were considered for this study: 333 isolates were recovered from non-diarrheal samples and 45 from diarrheal cases (defined as more than four liquid stools in a 24-hour period).^25^ The second study was conducted for 43 months in Santa Clara de Nanay, a rural community in Iquitos, located in the Amazonian region of Peru.^15,27^ Samples were collected quarterly to determine the carriage state in children and a single sample was cultured per every diarrhea episode. This prospective, community-based surveillance study included 442 children, of whom 174 subjects provided 83 *Campylobacter* isolates collected from non-diarrheal samples and 202 isolates from diarrheal samples.^27^ In the Santa Clara study, an increase over three unformed stools in a 24-hour period was defined as diarrhea (Table 1).^27^

**Table 1:** Demographic summary of the Amazon and the coastal regions

	Population (*n*)	Gender		Age (months)	Diarrheal samples (*n*)	Non-diarrheal samples (*n*)	Non-culturable (*n*)	Not *Campylobacter jejuni* (*n*)	Removed (*n*)
		Male (%)	Female (%)						
Coastal region	99	44.6	55.4	27.4 ± 17.77	45	333	109	6	35
Amazon region	174	42.4	57.6	29 ± 17.14	202	83	78	29	5
Total	273	–	–	28.2 ± 17.54	247	416	187	35	40

### Culture.

Fresh stool samples from children from the coastal region were transported in Cary Blair transport media and cultured on *Brucella* agar with defibrinated sheep blood and Butzler selective and growth supplement for *Campylobacter*.^25^ Feces from children living in the Amazon region were transported in glycerol-buffered saline (GBF). Culture was performed by inoculating 100 μL of GBF onto a 0.45-μm nitrocellulose filter overlying a Columbia blood agar; filter was removed after 30 minutes.^15^ Plates were incubated at 42°C for 48 hours in micro-aerobic conditions (5% O_2_, 10% CO_2_, and 85% N_2_). Suspicious colonies were Gram-stained and biochemically tested for identification of *Campylobacter*. *Campylobacter jejuni* and *Campylobacter coli* species discrimination was made by hippurate hydrolysis.^15,25^ All isolates were stored in peptone with 20% of glycerol at −80°C.

Archived isolates were revived on Columbia Blood Agar Base (Oxoid, Basingstone, Hampshire, England) plates with 5% calf blood supplemented with *Campylobacter* Selective Supplement (Blaser-Wang, Oxoid) following incubation for 24 to 72 hours at 42°C in a micro-aerobic environment.

### DNA extraction.

Bacterial DNA was extracted with a Wizard Genomic DNA Purification Kit (Promega, Madison, WI) following the manufacturer’s directions. The DNA concentration was adjusted to 250 ng/µL using a NanoDrop 1000 spectrophotometer (Thermo Fisher Scientific, Wilmington, DE).

### PCR identification.

A PCR reaction was performed on each isolate to confirm species before inclusion in CPS typing. Amplification of fragments of the *glyA* gene was used to discriminate *C*. *jejuni* from *C. coli* species.^28^ *Campylobacter* isolates that were confirmed as *C*. *jejuni* were solely considered for CPS typing.

### Capsular polysaccharide typing.

The multiplex PCR method developed by Poly and others,^21^ which is able to detect all 47 Penner serotypes, collapses the Penner serotypes into 35 CPS complexes.

PCRs were performed with AmpliTaq DNA polymerase FS (Applied BioSystems, Foster City, CA). Amplification conditions were set up for all primer sets as follows: denaturation at 94°C for 30 seconds, annealing at 56°C for 30 seconds, and extension at 72°C for 45 seconds for a total of 29 cycles. PCR products were analyzed by electrophoresis on 14-cm-long, as the minimum length, 2.5% agarose gels (Invitrogen, Carlsbad, CA) in 0.5× Tris-borate-EDTA (Invitrogen) buffer at 100 V for 3 hours and then visualized in a gel image digitizer GelDoc XRS plus (Bio-Rad, Hercules, CA). PCR amplicon sizes were determined by comparison with a molecular DNA 100-bp ladder (Bio-Rad).

### Pulse field gel electrophoresis.

Isolates from serial infections in a subject that demonstrated identical CPS types were selected for genotyping by pulse field gel electrophoresis (PFGE). The *C. jejuni* isolates were grown for 18 hours on Mueller–Hinton agar plates at 37°C under micro-aerobic conditions. Cells were harvested in phosphate saline buffer pH 7.4 and adjusted to an optical density at 600 nm of 1.8 ± 1. A total of 10 μL of proteinase K (Life sciences), 10 mg/mL, are added to 100 μL of the bacterial culture and 100 μL of low-melting-point agarose (Invitrogen), aliquoted into 100 μL plugs (Bio-Rad) and allowed to solidify for 15 minutes at 5°C. The plugs are then transferred to 50-mL conical tubes containing 20 mL of cell lysis buffer (50 mM Tris, 50 mM EDTA pH 8.0, 1% Sarcosyl, 0.1 mg of proteinase K/mL). Cell lysis was performed at 54°C for 30 minutes in a shaking water bath. Following lysis, the plugs were transferred to phosphate buffer saline, pH 7.4, and store at 4°C until further use. The bacterial DNA within the plugs were transferred into 500 μL of fresh restriction buffer with eight units *SalI* and *XhoI* or *BssHII* (New England Biolabs) and incubated overnight at 37°C. Plugs were loaded on a 1% agarose gel and ran on a contour-clamped homogeneous electric field apparatus (Bio-Rad) with a switch time of 2.2 to 17.6 seconds and a field strength of 6 V/cm for 23 hours. The gels were stained in ethidium bromide, visualized under UV light, and recorded on a Gel Doc (Bio-Rad).

### Statistical analysis.

Statistical analysis was conducted to calculate relative risk (RR) using demographic data and data obtained from molecular techniques using STATA version 13.1 (StataCorp, College Station, Texas).

## RESULTS

Culture and molecular identification was conducted on a total of 454 *C. jejuni* isolates obtained from 220 enrolled patients. Another 209 isolates were removed from the CPS typing analysis; 174 *Campylobacter* were non-culturable and 35 isolates were unable to be confirmed as *C*. *jejuni* species. The 454 *C. jejuni*–confirmed isolates were typed by multiplex PCR to discriminate on the basis of capsule type. Of those subjected to CPS typing by PCR, 184 (41%) of the isolates were from the rural community in Iquitos and 270 (59%) were from the peri-urban shantytown in Lima.

### Capsule type distribution.

**Figure 2.**
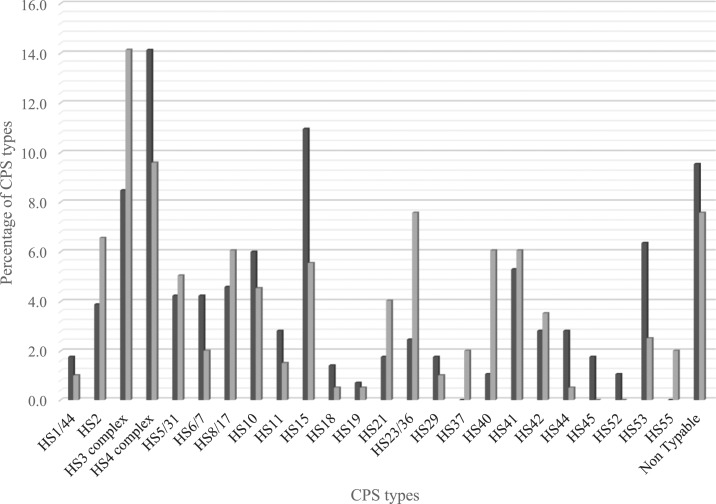
Percentage of capsular polysaccharide type distribution in the Amazon (black bars) and the coastal (gray bars) regions. Capsule types HS4 complex, HS3 complex, HS15, and HS41 represent the most frequent isolates in both regions.

A total of 24 different capsule types were discovered among the 454 strains. Overall, HS4 complex, HS3 complex, and HS15 were the most prevalent capsule types accounting for 12%, 11%, and 9% of the *C. jejuni* isolates, respectively (Figure 2). HS4 complex (14%) and HS15 (11%) were the most two common CPS types in isolates from the peri-urban samples collected near Lima (Figure 2). By contrast, HS3 complex (14%) was the most prevalent CPS type in those samples collected from the cohort near the Amazonian city of Iquitos. Capsular polysaccharide types HS45 and HS52 were found only in the peri-urban samples from Lima, whereas capsule types HS37 and HS55 were found only in the samples collected from the Amazon site near Iquitos (Figure 2).

### Demographic data analysis.

Next, we analyzed the RR of infection with *C*. *jejuni* with CPS types HS4 complex, HS3 complex, and HS15 because those CPS types are the most prevalent and their possible relationship with age and gender. Capsular polysaccharide type HS15 was associated with a 0.49 decreased risk to be infected with *C*. *jejuni* in males compared with females, adjusted by age (RR = 0.51, 95% CI: 0.28–0.95). For CPS complexes HS3 (RR = 1.38, 95% CI: 0.79–2.40) and HS4 (RR = 0.87, 95% CI: 0.46–1.36), there were no differences in infection rates between the genders. The risk of infection with *C*. *jejuni* increased with age for CPS type HS15 adjusted by gender (RR = 1.05, 95% CI: 1.01–1.09) and HS4 complex 0.78 (RR = 0.78, 95% CI: 0.64–0.95). HS3 demonstrated no difference in infection rates over the first years of life in this study.

### Capsular polysaccharide types and clinical features.

To evaluate whether there was a relationship between symptoms of enteric illness and capsule type carriage, we compared CPS type prevalence in patients with diarrhea (symptomatic) and without diarrhea (asymptomatic) (Table 2). No significant association was observed between clinical presentations (i.e., symptomatic versus asymptomatic) for carriage of the HS4 complex (RR = 1.19, 95% CI: 0.66–1.83), HS3 complex (RR = 1.32, 95% CI: 0.76–2.31), or HS15 (RR = 0.81, 95% CI: 0.45–1.51). Interestingly, we observed a significant association between isolates with a typeable CPS and the RR to become overtly ill by *C*. *jejuni* (RR = 0.70, 95% CI: 0.57–0.86). In other words, there was a greater likelihood of becoming ill if a subject was infected with a *C*. *jejuni* strain possessing any identifiable CPS type by multiplex PCR compared with a person infected by a *C*. *jejuni* with a non-typable CPS. Notwithstanding, this association existed only at the level of individual variable risk analysis, but no association was observed when the analysis was adjusted by age and gender.

**Table 2:** Comparison of capsular polysaccharide percentages between symptomatic and asymptomatic pediatric population

	Symptomatic		Asymptomatic	
Penner type	*n*	(%)	*n*	(%)
HS1/44	2	(1.2)	5	(1.6)
HS2	13	(8.0)	11	(3.4)
HS3 complex	18	(11.1)	34	(10.7)
HS4 complex	21	(13.0)	38	(11.9)
HS5/31	5	(3.1)	17	(5.3)
HS6/7	2	(1.2)	14	(4.4)
HS8/17	7	(4.3)	18	(5.6)
HS10	7	(4.3)	19	(6.0)
HS11	4	(2.5)	7	(2.2)
HS15	14	(8.6)	28	(8.8)
HS18	1	(0.6)	4	(1.3)
HS19	1	(0.6)	2	(0.6)
HS21	9	(5.6)	4	(1.3)
HS23/36	10	(6.2)	12	(3.8)
HS29	1	(0.6)	6	(1.9)
HS37	1	(0.6)	3	(0.9)
HS40	11	(6.8)	4	(1.3)
HS41	12	(7.4)	15	(4.7)
HS42	3	(1.9)	12	(3.8)
HS44	1	(0.6)	8	(2.5)
HS45	1	(0.6)	4	(1.3)
HS52	0	(0.0)	3	(0.9)
HS53	6	(3.7)	17	(5.3)
HS55	1	(0.6)	3	(0.9)
Non-typable	11	(6.8)	31	(9.7)
Total	162	(100)	319	(100)

From both locations, 146 of the 220 patients had multiple infections. In the rural Amazonian cohort, 60 children in this dataset experienced repeated episodes of *Campylobacter* infections, ranging from two to six episodes during the 4 years of study with the reinfection interval ranging from 1 week up to 180 weeks. We investigated the CPS type by multiplex PCR in 40 individuals with multiple infections. Of the individuals with repeated infections, 85% (*n* = 34) were caused by strains of different CPS types, whereas only six (15%) individuals experienced recurring infections with isolates of the same CPS type. For the peri-urban cohort near Lima, 86 patients had repeated infections with *C*. *jejuni*, from two to 15 infections within 15 months of the study; multiple infections varied from a minimum of 4 days to a maximum of 60 days reoccurring infections. Sixty-five of those patients had a subsequent *C. jejuni* infection, of which we were able to CPS type by PCR. Of these, 85% (*n* = 55) were infected by *C*. *jejuni* of a different capsule type each time (more than 5 weeks apart), whereas 10 (15%) individuals experienced recurring infections with isolates of the same CPS type.

Furthermore, to determine whether those multiple infections with the same CPS type were from the same strain, we genotyped the isolates by PFGE (Supplemental Figure 1). Among the Amazonian cohort samples, three of six patients presented similar profiles, which most likely represented carrier state/recrudescent cases. In the other three cases, isolates presented different genomic profiles and were discrete infection events with an identical CPS type. In addition, in the peri-urban cohort, eight patients suffered potential reinfections with isolation of the microorganisms with different genomic profiles varying from 4 to 31 weeks apart. Moreover, two of the eight patients suffered up to three reinfections that varied from 8 to 30 weeks apart. Globally, CPS typing and PFGE analysis of isolates from patients with multiple infections indicate that in this study, *C. jejuni* capsules potentially provided 92% (135/146) of protection against reinfection with *C*. *jejuni* with an identical CPS type.

**Figure 3.**
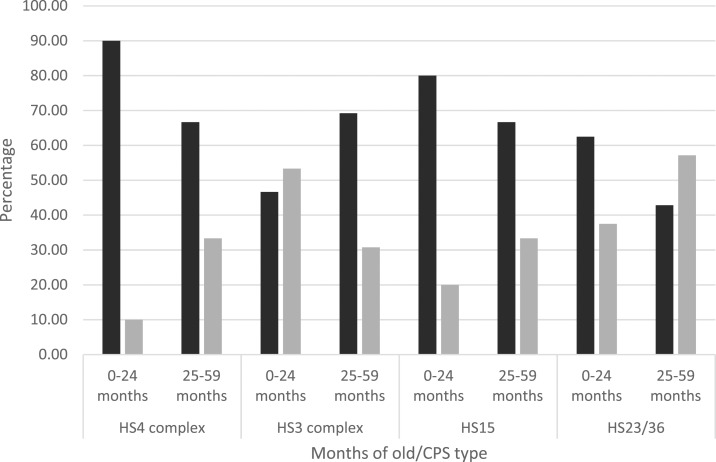
Comparative chart of age-related capsular polysaccharide (CPS) type prevalence for the most common Penner types in the Amazon region. Black bars indicate percentage of symptomatic cases, whereas gray bars indicate asymptomatic cases for CPS types HS4 complex, HS3 complex, HS15, and HS23/36.

**Figure 4.**
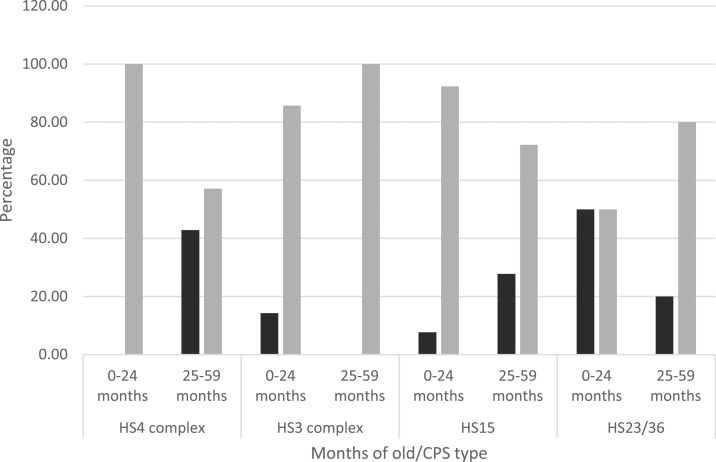
Comparative chart of age-related capsular polysaccharide (CPS) type prevalence for the most common Penner types in the coastal region. Black bars indicate percentage of symptomatic cases, whereas gray bars indicate asymptomatic cases for CPS types HS4 complex, HS3 complex, HS15, and HS23/36.

To evaluate the role played by CPS types during first months of life, we evaluated frequency patterns of the most common CPS types and its association with clinical symptomatology before and after 2 years of age. For that purpose, we divided both regions into two pediatric populations: group 1 included the first 24 months, whereas group 2 included 25–59 months of age. In the Amazon region, we observed a greater percentage of symptomatic infections for CPS types HS4 complex, HS15, and HS23/36 in the first group. Unlike the latter, the percentage of symptomatic infections was greater in the second group for HS3 complex (Figure 3). In the coastal region, the greater percentage of asymptomatic infections was observed in the first group for CPS types HS15 and HS4 complex, whereas the greater percentage of asymptomatic infections was observed in the second group for CPS types HS23/36 and HS3 complex (Figure 4). Nonetheless, differences in symptomatology between Amazon and coastal regions might be entirely related to sampling methodologies rather than susceptibility.

## DISCUSSION

*Campylobacter’s* CPS has become an attractive antigen in the development of a protective vaccine to prevent *C. jejuni* infections in humans. Questions regarding the distribution of CPS types, temporal stability, their association with clinical manifestations, and severity remain to be answered. However, this information is crucial to the development of an effective multivalent CPS conjugate vaccine. Nonetheless, serotyping of *C*. *jejuni* by means of Penner’s serotyping scheme has been mainly conducted in reference laboratories in developed countries.^20^ In this report, we used a PCR assay to type the CPS of 454 isolates of *C*. *jejuni* from two different geographical locations in Peru. These regions differ in weather environmental conditions but are similar in regard to poor sanitation systems, socioeconomic status, and direct contact of populations with poultry.^25,27^ The most prevalent CPS types in our study were HS4 complex (12.3%), HS3 complex (10.8%), HS15 (8.7%), HS41 (5.6%), HS10 (5.4%), HS8/17 (5.2%), and HS2 (5%) and accounted for 53% of the total number of infections in this pediatric population. Capsular polysaccharide complexes HS3 and HS4 were the most prevalent in both regions. Seven CPS types represent more than 50% of the typed isolates. Nearly 60% of isolates were represented by five CPS types HS2, HS4 complex, HS8/17, HS3 complex, and HS5/31 in a mixed population in South and Southeast Asia, where more than 70% of the isolates from children were represented by the same CPS types.^21^ Similar results were shown by Sainato et al.,^29^ in a pediatric population from Egypt. CPS types HS2, HS3 complex, HS15, HS4 complex, HS5/31, and HS6/7 represent 60% of the total isolates. In our study, however, modest geographic differences were observed in CPS type distribution. Variances in CPS distribution between the two study populations may be attributable to geographic differences, local environmental conditions, and/or design study. Notwithstanding, more studies are needed to further depict these similarities and disparities.

This study revealed that the most prevalent CPS types in the symptomatic population were HS4 complex, HS3 complex, HS15, HS2, HS41, HS40, HS23/36, and HS21. Whereas the dominant CPS types in the asymptomatic population were HS4 complex, HS3 complex, HS15, HS8/17, HS5/31, and HS53 (Table 2). In a symptomatic and asymptomatic children population in Bangladesh, a CPS type distribution analysis conducted by Islam et al.,^30^ dominant capsule types were HS5/31, HS3 complex, HS4 complex, HS8/17, HS1/44, and HS6/7 for the enteritis group, whereas the CPS types HS5/31, HS3 complex, HS4 complex, HS8/17, HS9, and HS6/7 were dominant in the control group. As it can be observed, CPS types HS4 complex and HS3 complex are both prevalent in all groups in both studies. Noteworthy, HS15 is solely present in our results in both groups.

Capsular polysaccharide typing by means of a PCR is considerably easier and faster to achieve than the classic Penner serology-based technique, and obviates the need for difficult to obtain highly specialized reagents, especially in resource-limited areas. The Penner serotyping technique relies on the interaction of a specific antibody and the CPS structure; therefore, the outcome depends directly on the expression of the CPS, which turns on and off variably. A systematic review of the globally circulating CPS types from 1978 to 2002 encountered a 14% of non-typable *C*. *jejuni* isolates by means of the serology technique.^20^ In contrast to other studies that have used the Penner serology technique where the percentages of non-typable isolates have been appreciably higher, only 9% of the tested isolates in this study were non-typable by the PCR. Similar results were found in a pediatric population from Egypt^29^ and a children population from the Netherlands and Bangladesh, where 11% and 11.4% of the isolates were non-typable, respectively. Notably, Garrigan et al.^31^ and Poly et al.^21^ found lower percentages of non-typable isolates, in 3% of the adult population in the United States and 2% of a mixed native and foreign population in South Asia and Southeast Asia, using the CPS typing multiplex PCR. Unlike the latter, the CPS typing technique yielded a higher percentage of non-typable isolates in a children population from Bangladesh nonetheless.^30^ The inability to type every isolate using this methodology is a limitation. Besides, this technique is not able to detect the exact sugar composition of CPS, including the presence or absence of *O*-methyl phosphoramidate (Me*O*PN) moieties,^21^ a molecule that plays critical biological roles in *C*. *jejuni*.^32^ However, with its full coverage of the Penner types and by grouping them into cross-reacting complexes, this PCR-based technique is a useful tool in garnering the epidemiological data necessary for the development of a multivalent vaccine.

There are few studies regarding CPS distribution in low-middle income countries with endemic diarrheal disease. The vast majority of studies have been conducted in developed countries where different Penner type distributions have been observed. A handful of studies have been conducted in Asia and Africa, whereas no data have been published from South America.^20^ Recent studies describing Penner types in developing and developed countries have shown that the most common CPS types differ by geographical locations and economical status as well.^20^ Notably, dominant Penner types in our study, HS4 complex, HS15, and HS3 complex, differ to those globally dominant serotypes, thus increasing our need to continue describing the CPS types circulating in endemic areas. To determine the valency of a future protective vaccine, it is critical to increase our knowledge about CPS type prevalence in endemic areas so that a significant amount of the susceptible population is protected. Our study reinforces the need for more surveillance studies to be conducted in developing countries to understand CPS type distribution.

In this study, most (92%) of the enrolled subjects who suffered reinfections were infected with a CPS type that was different from their prior infection, suggesting CPS type may confer some level of protection against future reinfections. This finding agrees with that of Miller et al.,^33^ showing relatively common serotypes (i.e., HS4 complex, HS2, and HS1/44) are more likely to infect children aged 0–4 years than older age groups. Similarly, Linneberg et al.^34^ have shown that IgG antibodies against *C*. *jejuni* increase with age.

Conversely, a number of enrolled subjects (8%) had repeat infections with organisms of the same CPS type. Similar to this, a study in immunocompetent volunteers demonstrated no homologous protection against the CG8421 *C*. *jejuni* strain, although increased serum and fecal IgG and IgA antibodies were observed after first infection.^35^ Moreover, a case report of an immunocompetent volunteer did not show any acquired immune response against reinfection by *C*. *jejuni*.^36^ Lack of protection against *C*. *jejuni* infections has been mainly observed in immunocompromised subjects and those at the extremes of age.^37^ Fimlaid and others obtained similar results when specific antibodies and cytokines were measured against a homologous rechallenge against *C*. *jejuni* CG8421.^38^

In addition, the genotyping of the isolates from children presenting with successive infection with an identical CPS corresponded to the same strain suggests a carrier state/recrudescence in our cohorts. Recrudescence of *C*. *jejuni* infection is not uncommon.^36^

However, several studies are needed to describe the specific role played by capsules in infectious disease and its recognition by the immune system to prevent reinfections by this microorganism in endemic communities. These data provide important implications for CPS-based conjugate vaccine development strategies by focusing potential multivalent vaccines against specific *C. jejuni* CPS serotypes in humans, highlighting the importance of further studies describing CPS type distribution.

As previously stated, CPS type distribution varies according to geography and economical status. Thus, a multivalent prototype CPS-conjugated vaccine must account for such differences and include the most prevalent CPS types to protect most of the at-risk population against infectious diarrhea. Nonetheless, the most common CPS types, HS2, HS4 complex, and HS1/44, represent more than a third of infections caused by *C*. *jejuni* globally,^19^ so a CPS-conjugated vaccine including those most prevalent CPS types and those regarding regional/economical variation would dramatically increase the proportion of risk population that might benefit from this prototype vaccine. Moreover, a prototype vaccine would prevent not only from disease but also from stunting and decreasing of the carriage state.^15,36^ However, for such a multivalent CPS-conjugate vaccine to become a reality, surveillance studies in hyperendemic countries are sorely needed to further assess the distribution and role of CPS types in disease and protection. To that end, the PCR-based technique used in this study may be a useful and practical tool in determining the relative prevalence and temporal stability of those most common CPS types circulating in hyperendemic areas and broaden our understanding of *C. jejuni* infections for the development of a successful vaccine.

## Supplemental materials

Supplemental figure
