# Supplementary figures and images for: Distribution of Capsular Types of Campylobacter jejuni Isolates from Symptomatic and Asymptomatic Children in Peru
Source: Am J Trop Med Hyg. 2019 Aug 5;101(3):541–8. doi: 10.4269/ajtmh.18-0994 (PMC6726929; doi:10.4269/ajtmh.18-0994)

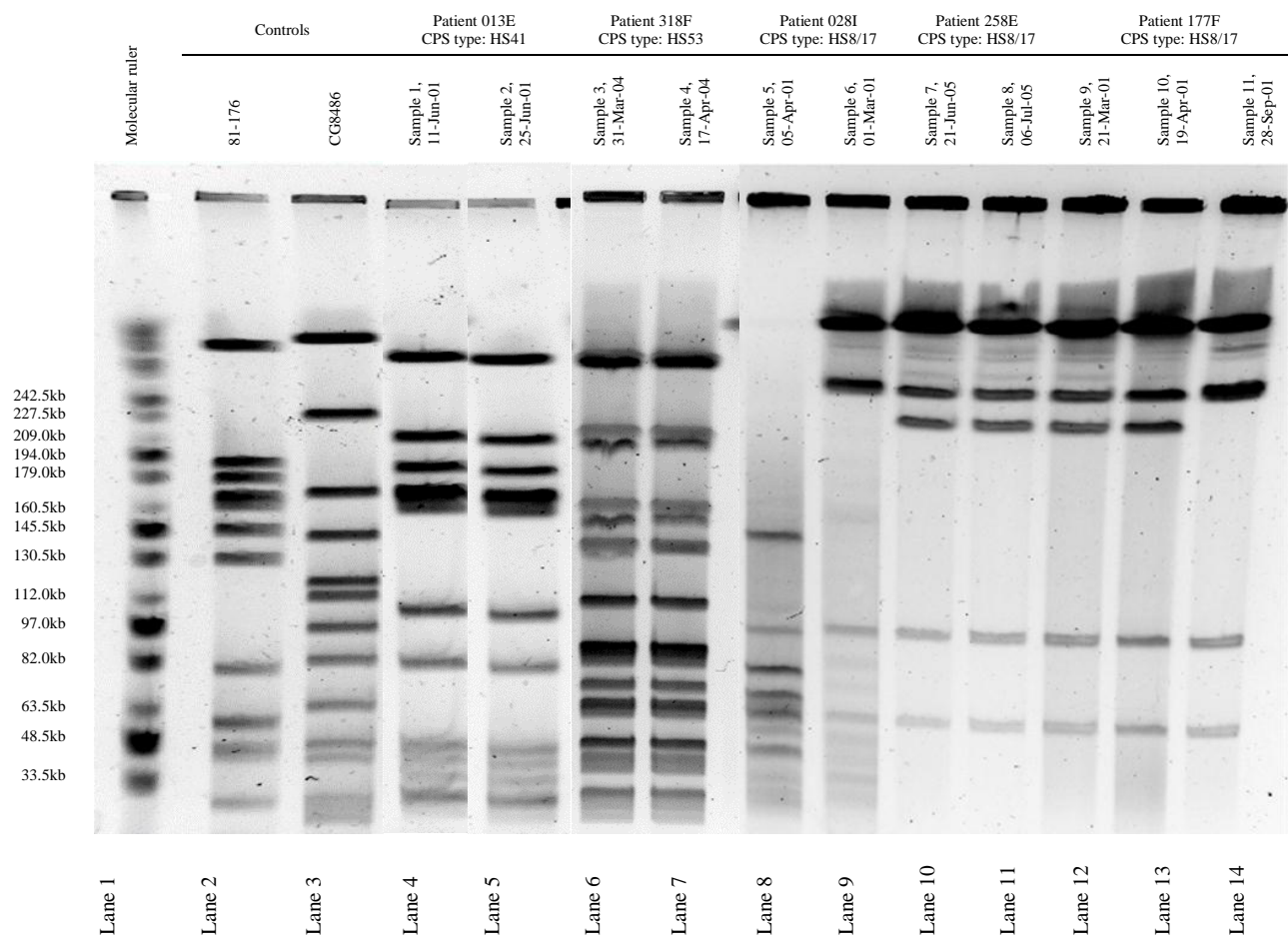

1

2 Figure S1. Illustration of PFGE gel.

Supplement: Supplementary file 1 [file tpmd180994.SD1.pdf]
